# Complete mitochondrial genome of the distinct red-eared slider (*Trachemys scripta* ssp., Testudines: Emydidae) in Korea

**DOI:** 10.1080/23802359.2021.1899866

**Published:** 2021-03-18

**Authors:** Jaehong Park, Seung-Min Park, Jae-I Moon, Yun-Ju Song, Jae Hyeok Choi, Ha-Cheol Sung, Dong-Hyun Lee

**Affiliations:** a School of Biological Sciences and Biotechnology Graduate School, Chonnam National University, Gwangju, Korea; b Department of Biological Sciences, College of Natural Sciences, Chonnam National University, Gwangju, Korea; c Research Center of Ecomimetics, Chonnam National University, Gwangju, Korea

**Keywords:** *Trachemys scripta* ssp., invasive species, mitochondrial genome, phylogenetic analysis

## Abstract

The complete mitochondrial (mt) genome of *Trachemys scripta* ssp. in Korea was sequenced and characterized. The mt genome is constituted of 37 genes (13 protein-coding genes, 22 transfer RNA genes and 2 ribosomal RNA genes) and a control region. Phylogenetic analysis based on the complete mt genome showed that the unidentified turtle had the mt genome closely related to that of *T. s. elegans*, though it had distinct morphology compared to *T. s. elegans*. This study can provide information for biogeographical studies and management plan for invasive species.

The red-eared slider (*Trachemys scripta*) is native to the United States and is the most common and abundant turtle on Earth (Parham et al. 2020). As the popularity of the turtles and their exports to other countries increased, so did the frequency of intentional releases to other ecosystems (Parham et al. 2013; Koo et al. 2019). International Union for Conservation of Nature (IUCN) designated this turtle as the invasive species (http://www.iucn.org). The invasive species can lead to hybridization with native species, causing genetic contamination of native species (Koo et al. 2020; Parham et al. 2020). An unidentified turtle was found in Gwangju, Korea. It had no red line on both sides of its head, no yellow lines on carapace, and no black spot, but irregular patterns on the plastron, which was different from general morphology of the red-eared slider (Buhlmann et al. 2008). Therefore, we sequenced the complete mt genome of the unknown turtle. Consequently, it had the mt genome of *T. s. elegans*, which is referred to as *T. s.* ssp. This can contribute to understanding the effect of the invasive species, especially *T. scripta*.

The *T. scripta* ssp. specimen was collected from Gwangju (35°10′28.1″N; 126°54′35.9″E), Korea, and the total genomic DNA was extracted from the tail using the DNeasy Blood & Tissue kit (Qiagen, Valencia, CA) according to the manufacturer’s protocol. The extracted DNA sample was deposited at the Museum of Wildlife, located in Research Center of Ecomimetics, Chonnam National University, Korea (Specimen accession number: 2020-RCE-TSSSP001; shcol2002@chonnam.ac.kr). The mt genome was analyzed by primer walking method (Supplementary table 1) and sequenced using Applied Biosystems 3730XL DNA Analyzer (Bionics, Seoul, Korea). The reads were aligned and the complete sequence was annotated by comparing GenBank data (FJ392294, KM216748, and KM216749).

The complete mt genome of *T. s.* ssp. is 16,807 bp in length deposited in GenBank (Accession number: MW122291), and contains 13 protein-coding genes, 22 transfer RNA (tRNA) genes, 2 ribosomal RNA (rRNA) genes, and a putative long non-coding control region. 12 protein-coding genes, 14 tRNA genes, and 2 rRNA genes are encoded in heavy strand, whereas 1 protein-coding gene (NADH dehydrogenase subunit 6) and 8 tRNA genes in light strand. The nucleotide composition of the *T. s.* ssp. mt genome (A = 34.3%, T = 27.0%, C = 25.9%, and G = 12.9%) is identical with that of *T. scripta* Canada (A = 34.3%, T = 27.0%, C = 25.9%, and G = 12.9%), and almost identical to that of *T. s. scripta* China (A = 34.2%, T = 27.0%, C = 25.9%, and G = 12.9%) and *T. s. elegans* China (A = 34.2%, T = 27.0%, C = 25.9%, and G = 12.9%). The sequence comparisons of *T. s. ssp.* with *T. scripta* Canada or *T. s. elegans* China indicated 99.8 or 99.7% sequence identity, respectively. However, the sequence identity between *T. s.* ssp. and *T. s. scripta* China is 99.3%. Furthermore, *T. scripta* Canada (Accession number: FJ392294) is referred to as red-eared slider (*T. s. elegans*) in GenBank data, even though there is no subspecies name. These data indicate that *T. s. ssp.* is closer to *T. s. elegans* than *T. s. scripta*.

To investigate the phylogenetic position of *T. s.* ssp., the complete mt genome sequences of 13 species in *Testudines* were extracted from GenBank and the phylogenetic tree was constructed using MEGA X software (Figure 1) (Kumar et al. 2018). The sequences were aligned using MUSCLE algorithm and tree building was performed using the maximum likelihood method and Tamura-Nei model with 1000 bootstrap replicates (Tamura and Nei 1993; Edgar 2004). It was found that every *T. scripta* species is clearly clustered in monophyletic manner and separated completely with other species. And like a sequence identity data, *T. s.* ssp. is in closer position with *T. s. elegans* and *T. scripta* (equal to *T. s. elegans*) than *T. s. scripta*. This analysis implies that *T. s.* ssp. which are different from general morphology (*T. s. elegans*) exist, though the reason is unknown. These data provide important molecular data for further biogeographical studies and can be utilized meaningfully to control ecological disturbance by *T. s. elegans* which is an invasive species in many countries including Korea. 

**Figure 1.**
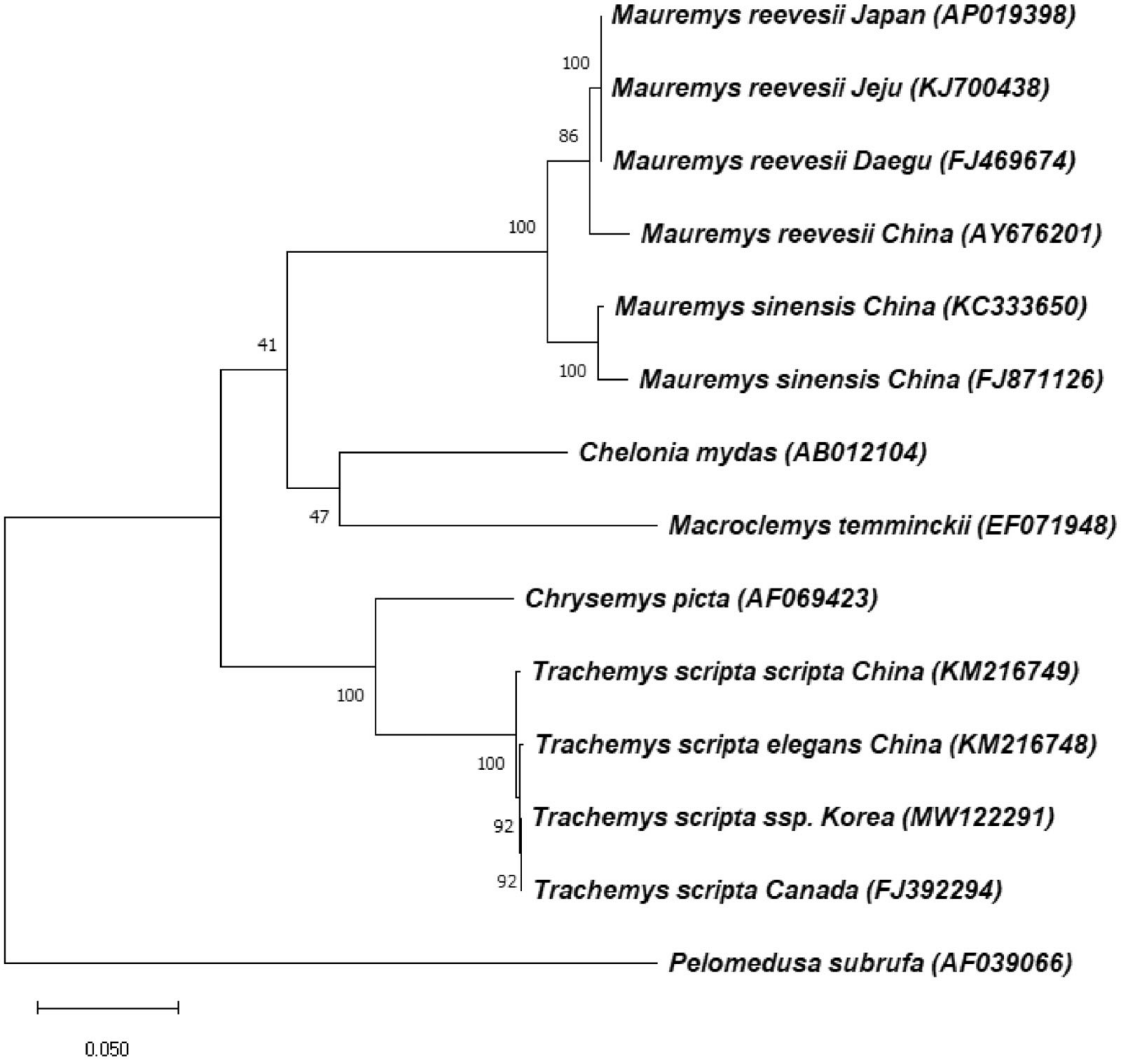
Phylogenetic tree of *Trachemys scripta* ssp. and other related species based on complete mt genome sequences. Phylogenetic analysis was performed using MEGA X software. GenBank accession numbers of each mt genome sequence are given in the bracket after the species name, and the bootstrap value based on 1000 replicates is represented on each node. *Pelomedusa subrufa* was used as outgroup to root the tree.

## Supplementary Material

Supplemental Material

## Data Availability

The data that support the findings of this study are openly available in NCBI at https://www.ncbi.nlm.nih.gov/nuccore/MW122291, reference number MW122291.

## References

[CIT0001] Buhlmann K, Tuberville T, Gibbons JW. 2008. Turtles of the southeast. Athens: University of Georgia Press.

[CIT0002] Edgar RC. 2004. MUSCLE: multiple sequence alignment with high accuracy and high throughput. Nucleic Acids Res. 32(5):1792–1797. 15034147 10.1093/nar/gkh340 PMC390337

[CIT0003] Koo KS, Baek H-J, Hwan KS, Jang H-J, Kim D-I, Sung H-C. 2019. First report on the Natural Movement of Introduced Turtle, *Trachemys scripta elegans*. Korean J Ecol Environ. 52(1):9–12.

[CIT0004] Koo KS, Song S, Choi JH, Sung HC. 2020. Current distribution and status of non-native freshwater turtles in the wild, Republic Of Korea. Sustainability. 12(10):4042.

[CIT0005] Kumar S, Stecher G, Li M, Knyaz C, Tamura K. 2018. MEGA X: molecular evolutionary genetics analysis across computing platforms. Mol Biol Evol. 35(6):1547–1549. 29722887 10.1093/molbev/msy096 PMC5967553

[CIT0006] Parham JF, Papenfuss TJ, Dijk PP, Wilson BS, Marte C, Schettino LR, Brian Simison W. 2013. Genetic introgression and hybridization in Antillean freshwater turtles (*Trachemys*) revealed by coalescent analyses of mitochondrial and cloned nuclear markers. Mol Phylogenet Evol. 67(1):176–187. 23353072 10.1016/j.ympev.2013.01.004

[CIT0007] Parham JF, Papenfuss TJ, Sellas AB, Stuart BL, Simison WB. 2020. Genetic variation and admixture of red-eared sliders (*Trachemys scripta elegans*) in the USA. Mol Phylogenet Evol. 145:106722. 31874235 10.1016/j.ympev.2019.106722

[CIT0008] Tamura K, Nei M. 1993. Estimation of the number of nucleotide substitutions in the control region of mitochondrial DNA in humans and chimpanzees. Mol Biol Evol. 10(3):512–526. 8336541 10.1093/oxfordjournals.molbev.a040023

