# Supplementary material for: Complete mitochondrial genome of the distinct red-eared slider (Trachemys scripta ssp., Testudines: Emydidae) in Korea
Source: Mitochondrial DNA B Resour. 2021 Mar 18;6(3):1077–9. doi: 10.1080/23802359.2021.1899866 (PMC7995828; doi:10.1080/23802359.2021.1899866)
Supplement: Supplemental Material [file TMDN_A_1899866_SM2845.pdf]

Supplementary Table. Information about primer sequences for PCR amplification

Primer sequences : Primers sequances and annealing temparatures used in PCR amplification.

For Peer Review Only

Primer names		Primer sequences (5'-3')	Annealing temperature (℃)
**1**	F	CTTATCATAAAGCACGGCAC	54
	R	CCTCAGGACCGGTTTAAATT	
**2**	F	GGTGTAGCTAACTGAGATGG	54
	R	TGGCTCAGTATTATTTGGGG	
**3**	F	ATGGAGGACAAAACACCAAA	54
	R	CTGAACTCAGATCACGTAGG	
**4**	F	ATCAACGAACTAAGCTACCC	54
	R	TATTATCAGCGGTCAGGATG	
**5**	F	AGTAGCTCAGACCATCTCAT	54
	R	ATTAGTGTTGAAGCAGTTGC	
**6**	F	ACAGTATCTAGCAACCACTG	54
	R	AGAAGGGTGACGGATAGTAT	
**7**	F	TTATAGCCACTGCAATAGCC	54
	R	GCTTCAATTCCTGATGATGC	
**8**	F	GTTGTCGTCACAGCCCAT	54
	R	AGTGGAAATGTGCAACTACA	
**9**	F	GGCGGAATAGTCAAATGAGA	54
	R	TTAGGTAGAGGACTCGTAGG	
**10**	F	ACCCTAGTACTCTACATCATCA	54
	R	GCTCATAGTTGGATGGTTGA	
**11**	F	CAATCCTACCAACAAAGACA	54
	R	TGTGATGTCCTTGGAAAGTT	
**12**	F	ACTTCAGGACTTGCCATATG	54
	R	ATTGCTACTCAGGAAGAACC	
**13**	F	TGCCATTACCCTATCTACAA	54
	R	TGTTTTCGTGGAATTGGTTC	
**14**	F	AACCTTCCCAAGAACTAACC	54
	R	AGCTAGCGAGTAGTCATCAT	
**15**	F	CACTTATAATCGCCCATGGA	53
	R	TCAGCGAGAATACTGTAGGA	
**16**	F	ACCCTTTGTGACGTAACTTT	53
	R	ACCTATGAGTGCTATGTTGC	
**17**	F	TAGCTTTCCTACACATCTGC	53
	R	GCCATCATTGAACTAGGACA	
**18**	F	ACCTCATTTCTCCTAACACT	53
	R	ATAGCCAAGAAAATTCCGGT	
**19**	F	CAATGACCATAAACCTCCGA	53
	R	GGGTTGTTCGTTGTTTTGAT	
**20**	F	CCTCCGCATATTAAACCAGA	53
	R	TGCTTATCTCTCGTGATTAGG	
**21**	F	TTCTACACCATGACTATCGC	53
	R	CTAGGTGTTTTGGGCTATCA	

For Peer Review Only
